# La polysérite tuberculeuse, une étiologie méconnue de thrombose portale: à propos d’un cas au Burkina Faso

**DOI:** 10.48327/mtsi.v5i4.2025.648

**Published:** 2025-12-01

**Authors:** Wendlassida Martin NACANABO, T. André Arthur SEGHDA, Anna TALL/THIAM, Yannick Laurent Tchenadoyo BAYALA, André K SAMADOULOUGOU

**Affiliations:** 1. Service de cardiologie, Centre hospitalier universitaire de Bogodogo, Ouagadougou, Burkina Faso; 2. Service de rhumatologie, Centre hospitalier Universitaire de Bogodogo, Ouagadougou, Burkina Faso

**Keywords:** Tuberculose extrapulmonaire, Polysérite tuberculeuse, Thrombose portale, Péricardite, Ouagadougou, Burkina Faso, Afrique subsaharienne, Extrapulmonary Tuberculosis, Tuberculous Polyserositis, Portal Vein Thrombosis, Pericarditis, Ouagadougou, Burkina Faso, Sub-Saharan Africa

## Abstract

**Introduction:**

La polysérite tuberculeuse constitue un défi diagnostique en raison de sa rareté et de sa gravité: elle peut entraîner des complications graves. La thrombose veineuse portale est une complication inhabituelle, souvent méconnue. Nous rapportons un cas clinique illustrant cette association chez un patient immuno compétent.

**Observation:**

Un homme de 31 ans, orpailleur avec notion de contage tuberculeux, est admis pour dyspnée, douleur thoracique, toux grasse, ainsi que des signes d’imprégnations tuberculeuses évoluant depuis deux mois. L’examen clinique objective une pleurésie gauche massive, une ascite modérée et un épanchement péricardique en pré-tamponnade. Léchographie abdominale met en évidence une thrombose partielle de la veine porte. Les analyses biologiques montrent une anémie inflammatoire, une leucopénie et des liquides d’épanchement exsudatifs. La présence du DNA de *Mycobacterium tuberculosis* est détectée par GeneXpert^®^ dans les prélèvements pleural et péritonéal. Le diagnostic de polysérite tuberculeuse compliquée de thrombose de la veine portale est retenu. Le patient est traité par une quadrithérapie antituberculeuse, un drainage pleural et péricardique, ainsi que par de l’acide acétylsalicylique pour sa thrombose veineuse portale. L’évolution est favorable avec une résolution clinique en deux mois et une guérison à six mois.

**Conclusion:**

Ce cas souligne l’importance de considérer la tuberculose dans les étiologies des thromboses veineuses inexpliquées, même chez un sujet immunocompétent. Un diagnostic précoce et une prise en charge adaptée permettent d’améliorer le pronostic.

## Abstract (en)

**Introduction:**

Due to its rarity and severity, tuberculous polyseritis is a diagnostic challenge that can lead to serious complications. Portal vein thrombosis is an uncommon complication that is often overlooked. This case report illustrates this association in an immunocompetent patient.

**Observation:**

A 31-year-old male gold miner with a history of tuberculosis infection was admitted with dyspnea, chest pain, a productive cough, and signs of tuberculosis impregnation that had developed over the course of two months. Physical examination revealed massive left pleurisy, moderate ascites, and pericardial effusion in the pre-tamponade stage. An abdominal ultrasound revealed partial thrombosis of the portal vein. Laboratory tests showed inflammatory anemia, leukopenia, and exudative effusions. *Mycobacterium tuberculosis* DNA was detected in pleural and peritoneal samples by GeneXpert. A diagnosis of tuberculous polyseritis complicated by portal vein thrombosis was made. The patient received four-drug antituberculosis therapy, as well as pleural and pericardial drainage and acetylsalicylic acid to treat his portal vein thrombosis. The outcome was favorable, with clinical resolution occurring in two months and full recovery occurring in six months.

**Conclusion:**

This case highlights the importance of considering tuberculosis as a possible cause of venous thrombosis, even in immunocompetent individuals. Early diagnosis and appropriate management improve the prognosis.

## Introduction

La tuberculose demeure un problème majeur de santé publique mondiale [5], particulièrement dans les pays en voie de développement comme le Burkina Faso [13] où sa prévalence reste élevée. Les manifestations extra-pulmonaires posent de véritables défis diagnostiques par leur polymorphisme clinique [13]. La polysérite tuberculeuse, une forme rare et grave de tuberculose extrapulmonaire, est souvent méconnue en raison de sa présentation clinique atypique [7]. Son spectre clinique est varié, allant de la pleurésie classique à des complications inhabituelles comme la thrombose de la veine porte [14] retardant ainsi le diagnostic [16]. L’analyse des liquides d’épanchement à la recherche du bacille de Koch se révèle habituellement peu contributive, retarde le diagnostic, ce qui impacte fortement le pronostic. Nous rapportons le cas d’un patient de 31 ans, immunocompétent, atteint d’une polysérite tuberculeuse compliquée d’une thrombose de la veine porte.

## Observation clinique

**Figure 1:**
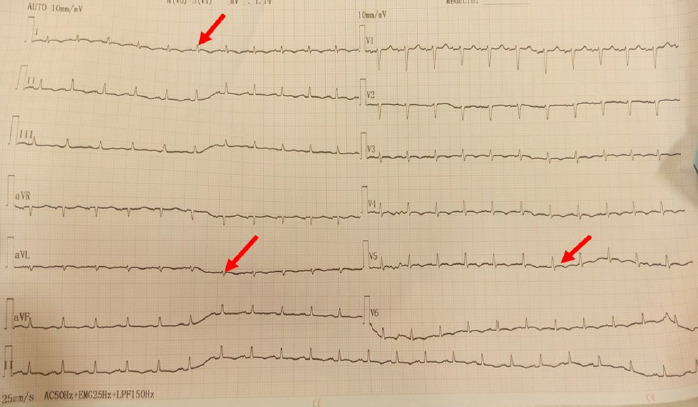
Électrocardiogramme de 12 dérivations montrant une tachycardie sinusale régulière à 112 cycles par minute, un bas voltage diffus et un trouble de la repolarisation à type onde T négative diffuse

**Figure 2:**
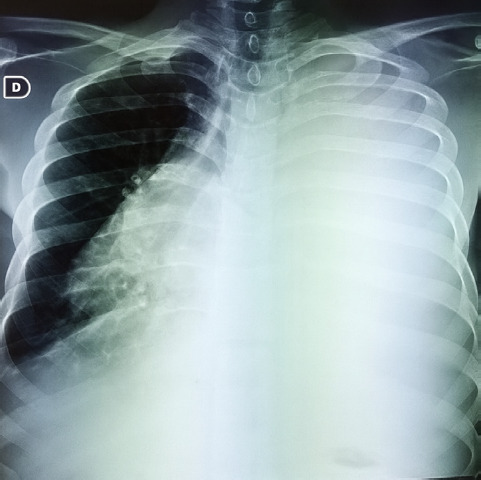
Radiographie thoracique de face montrant un épanchement pleural de grande abondance associé à une cardiomégalie

**Figure 3:**
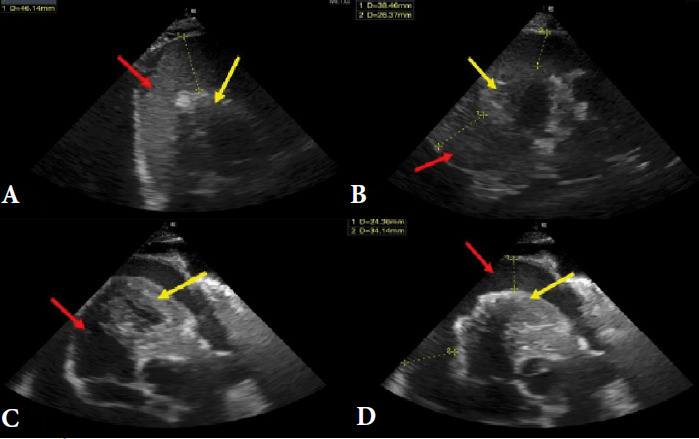
Échocardiographie transthoracique montrant A et B: épanchement péricardique de grande abondance (flèches rouges), compression des cavités cardiaques (flèches jaunes), C et D: après évacuation de deux litres de liquide péricardique, persistance d’un épanchement circonférentiel (flèches rouges) avec cavités cardiaques non comprimées (flèches jaunes)

**Figure 4:**
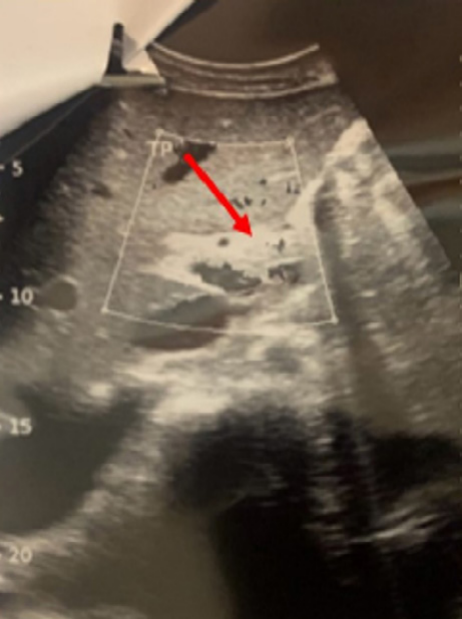
Échographie abdominale montrant une thrombose portale (flèche rouge)

Il s’agit d’un patient de 31 ans, orpailleur traditionnel, avec une notion de contage tuberculeux, sans autres antécédents pathologiques. Il a été admis pour dyspnée stade 3 et douleur thoracique. Cette douleur était permanente, d’intensité modérée, sans irradiation et calmée par la position penchée en avant. À cette symptomatologie se serait associée depuis deux mois une toux grasse avec expectorations muqueuses. Il existe un contexte d’asthénie, d’anorexie, d’amaigrissement, de sueurs nocturnes et de fièvre vespérale, une détresse respiratoire avec saturation à l’air ambiant à 78% et une fréquence respiratoire à 26 cycles par minute. L’examen clinique permettait de détecter un épanchement pleural liquidien gauche, une ascite de moyenne abondance et un assourdissement des bruits du cœur. La biologie notait une anémie inflammatoire à 9 g/dl, la formule sanguine une leucopénie 2 600/μl. La fonction rénale et hépatique étaient normales. Le liquide d’ascite comportait essentiellement des lymphocytes. La recherche de bacilles acido-alcoolo-résistants (Baar) et la culture dans le liquide pleural, péritonéal, dans les crachats et les urines étaient négatives. L’examen biochimique de ces liquides a mis en évidence un exsudat (taux de protéine > 30 g/l). L’intradermo-réaction tuberculinique était positive à 20 mm. Le test GeneXpert® du liquide pleural et péritonéal était positif, sans résistance à la rifampicine. Les sérologies des hépatites virales et des rétrovirus (VIH…) étaient négatives. Le dosage des marqueurs tumoraux tels que l’alpha fœto-protéine et l’antigène carcino-embryonnaire étaient normaux. L’électrocardiogramme réalisé en urgence notait une tachycardie sinusale régulière à 112 cycles par minutes, avec un bas voltage diffus et des ondes négatives (Fig. 1). La radiographie thoracique objectivait une opacité de tout le champ pulmonaire gauche avec déviation de la trachée à droite évocatrice d’une pleurésie gauche de grande abondance (Fig. 2). Par ailleurs, on notait la présence de cavernes de tailles variables au niveau apical du champ pulmonaire droit. L’échocardiographie Doppler notait une image hypoéchogène entourant le cœur qui était animé de mouvements pendulaires (de 42 mm en regard du ventricule gauche et de 30 mm en regard du ventricule droit) en rapport avec un épanchement péricardique circonférentiel de grande abondance en pré-tamponnade (Fig. 3). L’échographie abdominale montrait un foie d’échostructure normale, une ascite de moyenne abondance et une thrombose portale proche du hile hépatique, sans masse intra-abdominale (Fig. 4). Le diagnostic de polysérite tuberculeuse compliquée de thrombose veineuse portale était déjà présumé. Le patient a bénéficié d’une ponction pleurale d’urgence, d’une ponction d’ascite et d’une ponction péricardique, permettant de recueillir un liquide jaune citrin. Le traitement médicamenteux était constitué d’une quadrithérapie antituberculeuse faite de rifampicine, isoniazide, éthambutol et pyrazinamide pendant deux mois, suivie de l’association rifampicine et isoniazide durant quatre mois. De l’acide salicylique a été prescrit pendant six mois à cause de la contre-indication d’anticoagulant oral. L’évolution a été marquée par une régression complète des signes cliniques au bout de deux mois. Le patient a été réévalué six mois plus tard à la fin du traitement et a été déclaré guéri.

## Discussion

La tuberculose reste l’une des principales causes de morbidité et de mortalité dans les pays à revenu faible ou intermédiaire, tels que le Burkina Faso [3]. Selon l’Organisation mondiale de la Santé, les pays d’Afrique subsaharienne, dont le Burkina Faso, sont confrontés à une charge importante de la tuberculose, souvent compliquée par des formes multirésistantes [17]. La forme pulmonaire est la plus courante, cependant des cas de tuberculose extra-pulmonaire, notamment de polysérite, sont rencontrées, particulièrement chez les patients immunodéprimés [9,10,16]. Notre cas se distingue par sa survenue chez un sujet immunocompétent et associé à une complication grave. La prévalence des thromboses portales au cours des polysérites tuberculeuses reste peu étudiée du fait de la rareté des cas [2]. Cette atteinte pourrait être liée à une septicémie tuberculeuse; elle pourrait aussi être la conséquence d’hypercoagulabilité secondaire à l’inflammation chronique: elle perturberait la circulation sanguine portale. Elle augmente le risque d’hépatopathie et d’hypertension portale [6,8, 12]. Dans cette observation, le caractère exsudatif du liquide d’ascite est plus en faveur d’une infection tuberculeuse que d’une péritonite non infectieuse.

La gravité du tableau clinique de notre patient réside aussi dans la péricardite de grande abondance. Cette complication est fréquemment retrouvée dans des formes graves de la maladie. Elle peut évoluer vers une tamponnade cardiaque si elle n’est pas prise en charge rapidement [15]. Dans notre cas, la grande abondance de la pleurésie et de la péricardite s’expliquerait par une réponse inflammatoire chronique et un retard de traitement.

Le poumon « blanc », observé chez notre patient témoigne d’une atteinte pulmonaire diffuse, généralement liée à la miliaire tuberculeuse [4] qui reflète une dissémination hématogène de la bactérie et, bien que cette forme soit rare, elle reste l’une des causes de décès chez les patients non traités.

Pour établir avec précision le diagnostic de la tuberculose extra-pulmonaire on a recours à une combinaison de tests biologiques, radiologiques et parfois histopathologiques [1]. L’analyse radiologique joue un rôle important dans l’exploration de la plèvre et de l’épanchement péricardique. Les examens biologiques, tels que la culture de *Mycobacterium tuberculosis,* la PCR et la recherche de Baar dans les prélèvements, sont essentiels pour affirmer le diagnostic [11]. Dans notre cas, la confirmation de la tuberculose a reposé sur la positivité du GeneXpert^®^.

Le traitement de la polysérite tuberculeuse, comme d’autres formes de tuberculose extra-pulmonaire, repose principalement sur l’association de la rifampicine, de l’isoniazide, de la pyrazinamide et de l’éthambutol durant les deux premiers mois, suivie d’une phase de consolidation avec la rifampicine et l’isoniazide [5]. L’épanchement péricardique a nécessité un drainage pour soulager la pression sur le cœur et éviter une tamponnade. La thrombose portale n’a pas fait l’objet de traitement anticoagulant du fait du risque de complications hémorragiques inhérentes à l’épanchement péricardique de grande abondance.

Le pronostic de la polysérite tuberculeuse dépend largement de la rapidité du diagnostic et de la mise en place d’un traitement approprié [15]. Le retard dans le traitement des formes graves, comme celle observée dans notre cas, peut entraîner des défaillances organiques multiviscérales avec un pronostic défavorable [5]. Une surveillance prolongée et suffisamment étroite pour détecter des complications, comme l’épanchement péricardique et la thrombose portale, est cruciale pour éviter une dégradation clinique rapide.

## Conclusion

Ce cas clinique met en lumière la complexité du diagnostic et du traitement des formes atypiques de la tuberculose, d’où l’importance d’instaurer une approche multidisciplinaire pour mieux gérer les complications. Une sensibilisation sur la diversité clinique de cette infection, particulièrement dans les régions à forte endémie, est donc nécessaire pour permettre un diagnostic précoce et éviter sa dissémination.

## Approbation éthique et consentement à la participation

Nous avons obtenu le consentement éclairé du patient concerné. Toutes les mesures ont été prises pour préserver la confidentialité des informations le concernant.

## Financement

Aucun

## Contributions des auteurs et autrices

Wendlassida Martin NACANABO: conceptualisation, méthodologie, conservation des données, rédaction, révision et édition, rédaction de la version originale, administration du projet.

Taryètba André Arthur SEGHDA: administration du projet, investigation, méthodologie.

Yannick Laurent Tchenadoyo BAYALA: conceptualisation, investigation, conservation des données, administration du projet.

Anna TALL/THIAM: supervision, révision et édition

André Kounoaga SAMADOULOUGOU: super-vision, validation.

## Déclaration de liens d’intérêt

Aucun lien d’intérêt n’a été déclaré.
