# Supplementary material for: Tuberculous polyseritis, a little-known cause of portal vein thrombosis: a case report from Burkina Faso
Source: Med Trop Sante Int. 2025 Dec 1;5(4):mtsi.v5i4.2025.648. [Article in French] doi: 10.48327/mtsi.v5i4.2025.648 (PMC12948363; doi:10.48327/mtsi.v5i4.2025.648)
Supplement: Related texts

# Research article: Tuberculous polyseritis, a little-known cause of portal vein thrombosis: a case report from Burkina Faso

## Introduction

Tuberculosis remains a major global public health problem, particularly in developing countries such as Burkina Faso [13], where its prevalence remains high. Extrapulmonary manifestations pose real diagnostic challenges due to their clinical polymorphism [13].

One such manifestation is tuberculous polyseritis, a rare and severe form of extrapulmonary tuberculosis that is often overlooked due to its atypical presentation [7]. Its varied clinical spectrum ranges from classic pleurisy to unusual complications, such as portal vein thrombosis [14], thus delaying diagnosis [16]. Analyzing effusion fluids for Koch’s bacillus is usually unhelpful and delays diagnosis, significantly impacting prognosis. Here, we present the case of a 31-year-old immunocompetent patient with tuberculous polyseritis complicated by portal vein thrombosis.

## Clinical observation

The patient is a 31-year-old traditional gold miner with a history of tuberculosis infection and no other medical history. He was admitted due to stage 3 dyspnea and chest pain. The pain was constant and moderate in intensity. It did not radiate and was relieved by leaning forward. For two months, these symptoms had been accompanied by a productive cough with mucous sputum. The patient also presented with asthenia, anorexia, weight loss, night sweats, evening fever, and respiratory distress, with an oxygen saturation level of 78% and a respiratory rate of 26 cycles per minute. Physical examination revealed a left-sided pleural effusion, moderate ascites, and muffled heart sounds. Laboratory tests showed inflammatory anemia at 9 g/dL and leukopenia at 2,600/pL. Renal and hepatic functions were normal. The ascites fluid predominantly composed of lymphocytes. Tests for acid-fast bacilli (AFB) and cultures of pleural fluid, peritoneal fluid, sputum, and urine were negative. Biochemical analysis of these fluids revealed an exudate (protein level greater than 30 g/L). The tuberculin skin test was positive at 20 mm. The GeneXpert^®^ test of the pleural and peritoneal fluids was positive with no rifampicin resistance. Serology tests for viral hepatitis and retroviruses (HIV, etc.) were negative. Tumor marker levels, such as alpha-fetoprotein and carcinoembryonic antigen, were normal. An emergency electrocardiogram showed regular sinus tachycardia at 112 beats per minute with diffuse low voltage and negative waves (Fig. 1). The chest X-ray revealed opacity throughout the left lung and a deviation of the trachea to the right, indicating a significant amount of fluid in the left pleural cavity (Fig. 2). Additionally, cavities of various sizes were observed in the apical region of the right lung. Doppler echocardiography revealed a hypoechoic image surrounding the heart that was moving pendulously (42 mm opposite the left ventricle and 30 mm opposite the right ventricle), which is consistent with a large, circumferential pericardial effusion in the pre-tamponade stage (Fig. 3). An abdominal ultrasound showed a liver with a normal echotexture, moderate ascites, and a thrombosed portal vein near the hepatic hilum with no intra-abdominal masses (Fig. 4). A diagnosis of tuberculous polyseritis complicated by portal vein thrombosis was suspected. The patient underwent emergency pleural, ascites, and pericardial punctures, yielding a lemon-yellow fluid. The drug treatment consisted of a four-drug anti-tuberculosis regimen of rifampicin, isoniazid, ethambutol, and pyrazinamide for two months, followed by rifampicin and isoniazid for four months. Due to the contraindication of oral anticoagulants, salicylic acid was prescribed for six months. The patient’s condition improved significantly, with complete regression of clinical signs after two months. Six months later, at the end of treatment, the patient was reevaluated and declared cured.

**Figure 1:** 12-lead electrocardiogram showing regular sinus tachycardia at 112 cycles per minute, diffuse low voltage, and diffuse negative T wave repolarization disorder

**Figure 2:** Frontal chest X-ray showing a large pleural effusion associated with cardiomegaly

**Figure 3:** Transthoracic echocardiogram showing A and B: large pericardial effusion (red arrows), compression of the cardiac chambers (yellow arrows), C and D: after evacuation of two liters of pericardial fluid, persistence of circumferential effusion (red arrows) with uncompressed cardiac chambers (yellow arrows)

**Figure 4:** Abdominal ultrasound showing portal vein thrombosis (red arrow)

## Discussion

Tuberculosis remains one of the leading causes of morbidity and mortality in low-and middle-income countries, including Burkina Faso [3] . According to the World Health Organization, sub-Saharan African countries, including Burkina Faso, face a significant tuberculosis burden, which is often complicated by multidrug-resistant forms [17]. Pulmonary tuberculosis is the most common form, but cases of extrapulmonary tuberculosis, particularly polyseritis, are encountered, especially in immunocompromised patients [9,10,16]. Our case is notable for occurring in an immunocompetent individual and for being associated with a serious complication. The prevalence of portal vein thrombosis in tuberculous polyseritis is poorly understood due to the rarity of cases [2]. This condition may be associated with tuberculous septicemia or may result from hypercoagulability due to chronic inflammation, which disrupts portal blood circulation. It increases the risk of liver disease and portal hypertension [6,8,12]. In this case, the exudative nature of the ascites fluid is more consistent with a tuberculous infection than with non-infectious peritonitis.

The severity of our patient’s clinical presentation is also due to the significant pericarditis present. This complication is often seen in severe cases of the disease. If not treated promptly, it can progress to cardiac tamponade [15]. In our case, the significant pleurisy and pericarditis could be explained by chronic inflammation and delayed treatment.

The “white” lung observed in our patient is evidence of diffuse pulmonary involvement, which is generally associated with miliary tuberculosis [4] . This condition reflects hematogenous dissemination of the bacteria. Though rare, this form remains one of the leading causes of death in untreated patients.

Accurate diagnosis of extrapulmonary tuberculosis requires a combination of biological, radiological, and sometimes histopathological tests [1]. Radiological analysis is important for examining the pleura and pericardial effusion. Biological tests, such as culturing for *Mycobacterium tuberculosis,* PCR, and Baar’s test, are essential for confirming the diagnosis [11]. In our case, the diagnosis of tuberculosis was confirmed by a positive GeneXpert^®^ test.

Like other forms of extrapulmonary tuberculosis, the treatment for tuberculous pleurisy is mainly a combination of rifampicin, isoniazid, pyrazinamide, and ethambutol for the first two months, followed by a consolidation phase of rifampicin and isoniazid [5]. The pericardial effusion required drainage to relieve pressure on the heart and prevent tamponade. We did not treat portal vein thrombosis with anticoagulants due to the risk of hemorrhagic complications inherent in large pericardial effusions.

The prognosis for tuberculous polyserositis largely depends on the speed of diagnosis and initiation of appropriate treatment [15]. Delaying treatment of severe cases, as observed in our patient, can lead to multiorgan failure and a poor prognosis [5]. Close monitoring over a prolonged period is crucial to detect complications such as pericardial effusion and portal vein thrombosis and prevent rapid clinical deterioration.

## Conclusion

This clinical case underscores the difficulty of diagnosing and treating atypical forms of tuberculosis, which emphasizes the importance of a multidisciplinary approach to managing complications more effectively. Recognizing the clinical diversity of this infection, particularly in highly endemic regions, is essential for enabling early diagnosis and preventing its spread.

## Ethical approval and consent to participate

We obtained ethical approval and informed consent from the patient. All measures were taken to preserve the confidentiality of information concerning the patient.

## Funding

None.

## Authors’ contributions

Wendlassida Martin NACANABO: conceptualization, methodology, data preservation, writing, revision and editing, writing of the original version, project administration.

Taryètba André Arthur SEGHDA: project administration, investigation, methodology.

Yannick Laurent Tchenadoyo BAYALA: conceptualization, investigation, data curation, project administration.

Anna TALL/THIAM: supervision, review, and editing.

André Kounoaga SAMADOUOUGOU: supervision and validation.

## Conflicts of interest

The authors declare no conflicts of interest.
